# Human Mesenchymal Stromal Cells Attenuate Hyperoxia-Induced Cellular Impairment of Immature Oligodendrocyte and Neurons

**DOI:** 10.3390/cells15161493

**Published:** 2026-08-19

**Authors:** Meray Serdar, Karina Kempe, Josephine Herz, Francesca Ricci, Ursula Felderhoff-Müser, Ivo Bendix

**Affiliations:** 1 Department of Pediatrics I/Neonatology & Experimental Perinatal Neurosciences, Centre for Translational Neuro- and Behavioural Sciences (C-TNBS), University Hospital Essen, University Duisburg-Essen, 45147 Essen, Germany; 2 Global R&D, Chiesi Farmaceutici S.p.A., 43122 Parma, Italy; f.ricci@chiesi.com

**Keywords:** hyperoxia, immature oligodendrocytes, hippocampal neurons, human MSCs, hypoxic, proliferation, degeneration, differentiation, mitochondrial function

## Abstract

Preterm infants are at high risk of developing long-term brain injury such as encephalopathy of prematurity (EoP). Hyperoxia is a major contributor to EoP, affecting white and grey matter, with immature oligodendrocytes and hippocampal neurons being particularly vulnerable. While no causal therapy is available, mesenchymal stromal cells (MSCs) show therapeutic potential and are considered a promising candidate, although their effector mechanisms remain incompletely understood. Primary oligodendrocytes were isolated from mixed glial cultures of P0–P2 rats and hippocampal neurons from E16 rat embryos. On day 3 (oligodendrocytes) and day 5 (neurons) after isolation, cells were exposed to hyperoxia for 8 h and subsequently co-cultured indirectly with naive or hypoxic-preconditioned human MSCs (hMSCs) for 48 h under standard culture conditions. Degeneration, proliferation, differentiation and mitochondrial respiration were assessed in both cell types. Both naive and hypoxic-preconditioned hMSCs attenuated hyperoxia-induced degeneration, reduced proliferation and mitochondrial respiration failure. Although oligodendrocyte differentiation, assessed by myelin basic protein (MBP) expression, was modulated neither by hyperoxia nor by hMSC treatment, the dendritic structure in hippocampal neurons was impaired by hyperoxia and improved by hMSC treatment. Notably, hypoxic-preconditioned hMSCs showed a stronger therapeutic effect than naive hMSCs on hyperoxia-damaged hippocampal neurons. These findings indicate that indirect hMSC co-culture mitigates hyperoxia-induced impairment of immature oligodendrocytes and hippocampal neurons and that hypoxic preconditioning may modulate this effect in a cell type-specific manner.

## 1. Introduction

Worldwide, 11% of newborns are born premature, representing the largest patient cohort in paediatrics [1,2]. Despite increasing survival rates due to improved neonatal intensive care, the development risk of long-term injury such as encephalopathy of prematurity (EoP) is very high [3]. The immature brain is very sensitive to various noxious agents, such as increased oxygen concentrations (hyperoxia). At birth, there is a sudden rise in the partial pressure of oxygen, which affects the immature organism of a premature infant and is frequently potentiated by additional respiratory support [4,5]. Clinical trials demonstrated an inverse relationship between target oxygen saturation and outcomes in preterm infants. Higher target oxygen saturation reduces mortality but increases the risk of retinopathy, while lower target oxygen saturation shows the reverse pattern [6,7].

Clinical and experimental studies demonstrated changes in the white and grey matter, which can result in long-term disabilities such as motor-cognitive deficits and neuropsychiatric disorders [8,9,10]. Recent findings from clinical magnetic resonance imaging (MRI) studies at term-equivalent age suggested that adverse neurodevelopmental outcome in preterm infants can be primarily attributed to disturbed glial maturation, impaired neuronal connectivity and reduced cortical grey matter volume [11,12]. Experimental murine models of hyperoxia-induced EoP showed that, particularly, immature oligodendrocytes are affected [13,14,15,16,17]. Due to an immature antioxidant defence system, hyperoxia-induced production of reactive oxygen species (ROS) disturbs mitochondrial function and impairs cellular respiration [4,13,18,19,20,21,22,23,24,25,26,27], resulting in reduced ATP production, DNA damage, and release of pro-apoptotic factors that promote cell death and inhibit proliferation and differentiation, ultimately leading to oligodendrocyte degeneration and hypomyelination [4,13,18,19,27,28,29]. Furthermore, neonatal hyperoxia affects immature hippocampal neurons, resulting in degeneration and disturbed neurogenesis [16,17,30,31].

Despite extensive research, there is no causal therapy available for EoP. Recent advances in stem cell therapy offer promising potential for neuroprotection. Mesenchymal stromal cells (MSCs) have demonstrated strong positive effects in several diseases, including neonatal brain injury [32,33,34,35,36]. Previous studies have shown that MSC treatment can modulate oxidative stress and mitochondrial dysfunction [32,37], and both are critically involved in the pathophysiology of hyperoxia-induced brain injury [4,38]. In particular, hypoxic preconditioning has been shown to enhance the therapeutic efficacy of MSCs [39,40]. Under low-oxygen conditions (2–5% O_2_, hypoxia), MSCs show an improved differentiation potential, increased secretion of protective factors and enhanced survival under stressful conditions [39,40,41].

The central nervous system (CNS) has a high energy demand produced by mitochondria [42], and MSCs have been shown to improve mitochondrial function and cellular resilience under conditions of oxidative stress [39,43]. However, it remains unclear whether mitochondrial respiration is impaired in immature oligodendrocytes and hippocampal neurons during the recovery phase following hyperoxia. Furthermore, the potential therapeutic impact of hMSCs, and specifically hypoxic-preconditioned hMSCs, on the mitochondrial function has not been elucidated yet.

This study investigated effects of naive versus hypoxic-preconditioned hMSCs on primary immature oligodendrocytes and hippocampal neurons following hyperoxia. By using an in vitro co-culture model, we assessed the impact on cell viability, proliferation, differentiation and mitochondrial function, to determine whether hypoxic preconditioning enhances therapeutic effects of hMSCs.

## 2. Materials and Methods

### 2.1. Primary Cell Culture

All tissue collections were approved and performed in accordance with the guidelines of the University Hospital Essen, Germany, and with local government approval by the State Agency for Nature, Environment and Consumer Protection North Rhine Westphalia, Germany (approval number G1835/21; §04 TSchG).

#### 2.1.1. Human Mesenchymal Stem Cells (hMSCs)

Human Wharton’s jelly-derived mesenchymal stromal cells (hMSCs) were kindly provided by Chiesi Farmaceutici (Parma, Italy). According to the supplier, the cells were derived from donated umbilical cord tissue, isolated using a patented method (WO 2019/123407) and obtained with parental informed consent for medical research from a single donor. The hMSCs met the release criteria set by the International Society for Cell and Gene Therapy (i.e., more than 90% of the MSCs are CD73, CD90, and CD105 positive) and were tested to ensure sterility (i.e., negative for bacteria, fungi, mycoplasma, and endotoxin) [44,45].

Cells were used at passage 4 and cultured following a standardised protocol provided by the company. Briefly, frozen cells were thawed at 37 °C, centrifuged at 500× *g* for 10 min at room temperature (RT) followed by culture in hMSCs CHE2 medium (also provided by Chiesi Farmaceutici S.p.A.), containing 2000 U/L heparin and 5% human platelet lysate (from Chiesi Farmaceutici S.p.A.) at a density of 1.125 million per T75 flask at 37 °C, 5% CO_2_, and 95% humidity for 48 h. Prior to use, the cells were washed with 1× PBS (phosphate buffered saline), harvested using 3 U/mL trypsin/EDTA (Ethylenediaminetetraacetic acid; Gibco^®^Thermo Fisher Scientific, Waltham, MA, USA), centrifuged at 300× *g*, and counted using the cell counter NucleoView. For co-culture experiments with immature oligodendrocytes or hippocampal neurons, hMSCs were embedded in TrueGel3D hydrogel (TRUE1, Sigma-Aldrich, St. Louis, MO, USA), providing a 3D environment mimicking their native microenvironment more closely than conventional 2D culture [46]. This approach additionally allowed the separation of hMSCs from the target cells during the co-culture period, enabling a well-controlled, non-contact co-culture system. hMSC-containing hydrogels were plated onto transwell inserts (0.4 μm pore size filter; Constar, Corning, NY, USA) for 24 h under standard culture (hMSCs naive) or hypoxic conditions (1% O_2_, 37 °C; hMSCs hypoxic). This hypoxic preconditioning protocol was adopted from De Palma et al. 2025, where the same hMSC batch was used and cultured under the same hypoxic conditions, revealing a robust hypoxic response, including HIF-1α (hypoxia-inducible factor 1α) stabilisation and proteomic changes associated with a pro-regenerative secretory profile of preconditioned hMSCs [47].

#### 2.1.2. Oligodendrocyte Precursor Cells/Immature Oligodendrocytes (OPC)

Neonatal rat mixed glia cells were isolated from P0-P1 Wistar rats as previously described [13,14,48]. The cells were cultured in Dulbecco’s modified Eagle’s medium (DMEM, Gibco, Schwerte, Germany), supplemented with 20% foetal calf serum and 1% penicillin/streptomycin. After 10–12 days, immature oligodendrocytes, which had attached to the astrocyte layer of the mixed glia culture, were isolated by shaking overnight at 230 rpm. The collected cells were pre-plated for 30 min at 5% CO_2_ and 37 °C to remove contaminating microglia and astrocytes. Floating cells were collected, resuspended in proliferation medium (DMEM, 1× B27 (Gibco), 10 ng/mL platelet-derived growth factor (PDGF, PanBiotech, Aidenbach, Germany), 10 ng/mL basic fibroblast growth factor (bFGF, PanBiotech)), and plated onto poly-DL-ornithine-coated (Sigma-Aldrich) coverslips at a density of 100,000 cells/mL. Oligodendrocyte precursor cells were cultured in proliferation medium for 3 days before starting experiments.

Further treatment, including exposure to hyperoxia and co-cultivation with hMSCs, is described in detail in Section 2.2.

#### 2.1.3. Embryonic Hippocampal Neurons

Embryonic hippocampal neurons were isolated according to Salazar et al. [49]. Briefly, E16 embryos were collected from dams euthanised with 100 mg/kg ketamine and 10 mg/kg xylazine by caesarean section. The hippocampi were carefully dissected from the cerebrum and enzymatically digested with trypsin (2.5%, Sigma-Aldrich) and DNase (1%, Sigma-Aldrich). After enzymatic digestion, the hippocampi were mechanically disrupted using a glass pipette, and the total cell count was determined using the NucleoView cell counter. A total of 100,000 cells/mL were seeded onto poly-L-lysine-coated (Sigma-Aldrich, St. Louis, MO, USA) cover slips in neurobasal medium (NB, Gibco) supplemented with 1× B27, 1% glutamine, and 1% penicillin/streptomycin for 5 days before starting the experiments.

Details on exposure to hyperoxia and co-culture with hMSCs are provided in Section 2.2.

### 2.2. Study Design

**Figure 1:**
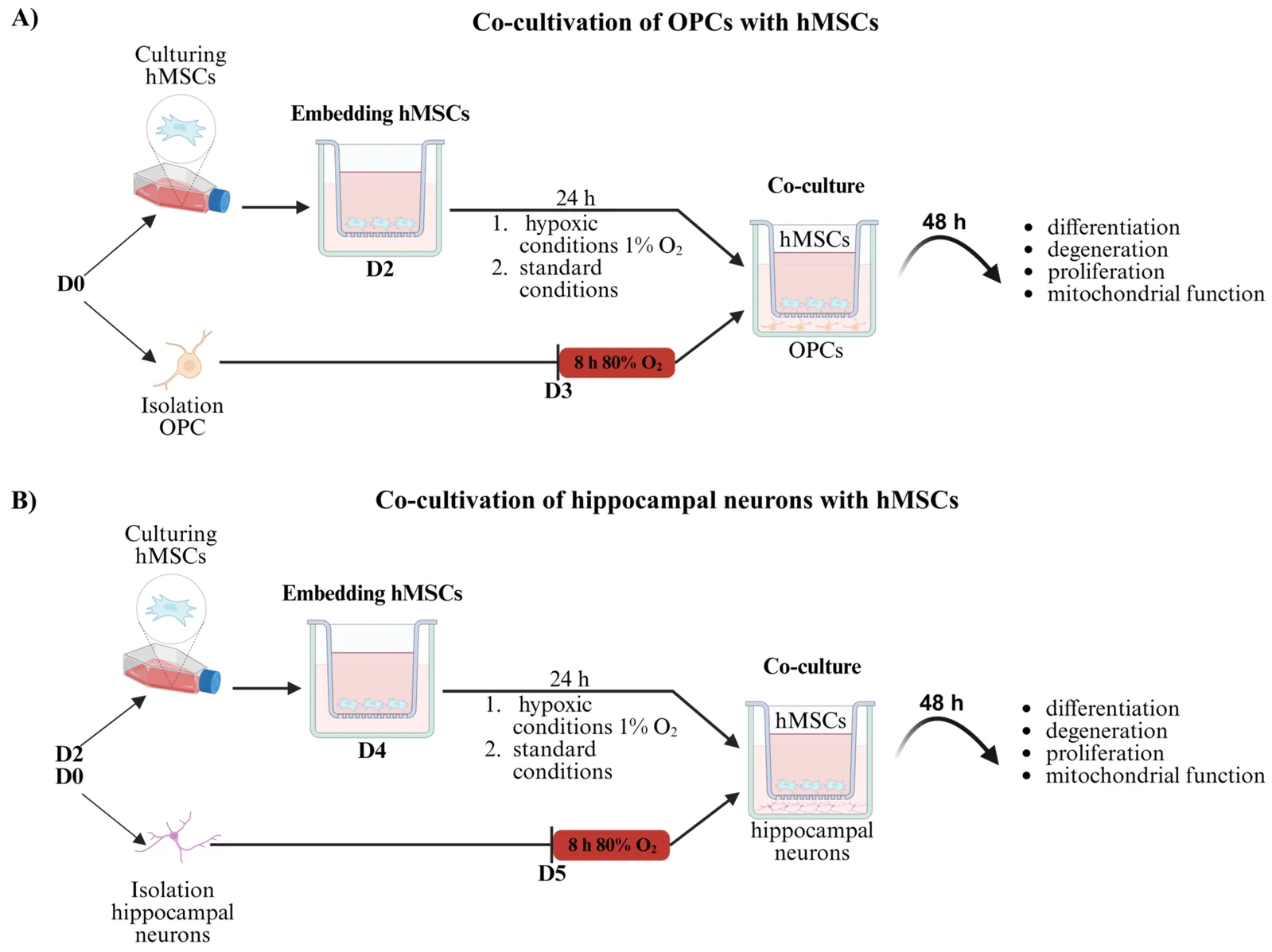
Experimental setup of indirect co-culture of OPCs and hippocampal neurons with naive or hypoxic-preconditioned hMSCs following hyperoxia exposure. (**A**) Primary oligodendrocyte precursor cells (OPCs) were exposed to hyperoxia (80% O_2_) for 8 h followed by indirect co-culture with naive or hypoxic-preconditioned hMSCs for 48 h. hMSCs were precultured under either standard or hypoxic conditions (1% O_2_) for 24 h prior to co-culture. (**B**) Primary hippocampal neurons were exposed to hyperoxia (80% O_2_) for 8 h followed by indirect co-culture with naive or hypoxic-preconditioned hMSCs for 48 h. Functional and morphological analyses of OPCs and hippocampal neurons included proliferation, degeneration, differentiation, and mitochondrial function. Figure was created in BioRender. J.H:. (2026); https://BioRender.com/0qcte9c; https://BioRender.com/llxlbh3.

Co-culture experiments with naive or hypoxic hMSCs and oligodendrocytes or embryonic hippocampal neurons are depicted in Figure 1. After 48 h, hMSCs were embedded in a gel-filled insert and subsequently cultured either under standard conditions or under hypoxic conditions for an additional 24 h. This 24 h period coincided precisely with the end of hyperoxia.

Following 3 days in culture, OPCs were exposed to hyperoxia (80% O_2_) for 8 h. Co-culture with naive or preconditioned hMSCs was started directly at the end of hyperoxia (Figure 1A). Embryonic hippocampal neurons were isolated at D0, and due to the 5-day culture duration, hMSC culture was started on D2, and co-culture was started on D5 accordingly (Figure 1B). After 48 h of co-culture, target cells were either fixed with 2% paraformaldehyde (PFA) for immunocytochemistry (ICC) analysis or further processed for assessment of mitochondrial metabolism. No medium change was performed during the 8 h hyperoxia exposure or the 48 h recovery/co-culture phase. The following six groups were analysed: (1) 21% O_2_ (normoxia), used here as a standard cell culture control condition rather than reflecting the physiological oxygen tension in the developing brain; (2) 21% O_2_ + MSC naïve; (3) 21% O_2_ + MSC hypoxic; (4) 80% O_2_ (hyperoxia); (5) 80% O_2_ + MSC naïve; and (6) 80% O_2_ + MSC hypoxic.

### 2.3. Immunocytochemistry

Following fixation, cells were incubated with blocking solution (5% normal goat serum in 0.1% Triton X-100 in PBS) for 1 h at room temperature followed by incubation with primary antibodies in PBS with 5% goat serum at 4 °C overnight. The following antibodies were used: anti-oligodendrocyte transcription factor 2 (Olig2; polyclonal rabbit anti-Olig2, 1:500; monoclonal mouse anti-Olig2, 1:300, Millipore, Darmstadt, Germany), anti-proliferating cell nuclear antigen (PCNA; polyclonal rabbit anti-PCNA, 1:800, Cell Signaling Technology, Frankfurt/Main, Germany), anti-myelin-basic-protein (MBP; monoclonal mouse anti-MBP, 1:500, Covance, Germany), anti-microtubule-associated protein 2 (MAP2; polyclonal mouse anti-MAP2, 1:500, Sigma-Aldrich), anti-X-linked doublecortin (DCX; polyclonal rabbit anti-DCX, 1:500, Cell Signaling Technology) and anti-betaIII-Tubulin (monoclonal mouse anti- betaIII-Tubulin, 1:500, abcam, Cambridge, UK). Specific antibody binding was visualised via incubation with the appropriate secondary antibodies (anti-mouse Alexa Fluor 555; anti-mouse Alexa Fluor 488; anti-rabbit Alexa Fluor 555; anti-rabbit Alexa Fluor 488; anti-rabbit Alexa Fluor 647, Invitrogen, Darmstadt, Germany; all 1:1000) for 1 h at room temperature. Nuclei were counterstained with Hoechst 33342 (1 µg/mL). Cover slips were mounted onto glass slides with DAKO Fluorescent Mounting Medium and kept in the dark at 4 °C. Degeneration was determined by staining of DNA fragmentation using terminal transferase dUTP nick end labelling (TUNEL) according to the manufacturer’s protocol (In situ Cell Death Detection Kit, Roche, Basel, Switzerland) in combination with Olig2 or betaIII-Tubulin. Cells were assessed via confocal imaging (A1plus, Eclipse Ti, with NIS-Elements AR software v2.2, Nikon, Düsseldorf, Germany, four laser lines (laser diode 405 nm, Ar laser 514 nm, G-HeNe laser 543 nm, Rn laser 639 nm)). Confocal z-stack (10 µm thickness, z-plane distance 2 µm) large-scale images of the cover slips were acquired with a 10 × objective.

Eight regions of interest (ROI, each 0.1369 mm^2^) were randomly selected and analysed by investigators blinded to treatment. The numbers of proliferating (PCNA^+^), degenerating TUNEL (TUNEL^+^), total oligodendrocytes (Olig2^+^) and hippocampal neurons (betaIII-Tubulin^+^) were quantified. The percentages of proliferating and degenerating oligodendrocytes as well as hippocampal neurons were determined. The differentiation capacity of oligodendrocytes was analysed by measuring the MBP^+^- area. Additionally, the areas of immature hippocampal neurons (DCX^+^) as well as dendrite area (MAP2^+^) were assessed. The differentiation capacity was further analysed by quantifying the terminal endpoints and dendrite lengths. For this, images of MAP2^+^- neurons were skeletonised, and the terminal endpoints of selected neurons were quantified manually. The total dendrite length of the selected MAP2^+^- neurons was automatically calculated by the NIS-software. For each independent cell preparation, values obtained from eight ROIs were averaged to generate a single value, representing one independent experiment. For dendritic morphology analyses, five neurons were randomly selected within each of eight ROIs per independent cell preparation (i.e., 40 neurons per independent experiment). These measurements were averaged to obtain one value per independent cell preparation/experiment, which served as the biological replicate for statistical analysis.

### 2.4. Oxygen Consumption Rate Measurement

The oxygen consumption rate was measured using Seahorse XF Cell Mito Stress Kit (103015-100, Agilent, Waldbronn, Germany) and sensor cartridges (102342-100, Agilent) per the manufacturer’s instructions. Injection ports of sensor cartridges were loaded with 5 µM Oligomycin, 1.25 µM carbonyl-cyanide-4-(trifluoromethoxy) phenylhydrazone (FCCP), and 5 µM Rotenone/Antimycin A (Rot/AA). The sensors were calibrated using a calibration plate. The oxygen consumption rate was measured for 90 min (immature oligodendrocytes) or 120 min (hippocampal neurons). Wave desktop 2.4 software (Agilent) was used to calculate the basal, ATP-linked, maximal, spare capacity and non-mitochondrial respiration (Supplementary Figure S1). All parameters were normalised to the number of nuclei. Therefore, cells were fixed and stained with Hoechst (see above) and counted automatically by using Image J 1.52a software (Java 1.8.0). Three independent experiments were performed, each including three technical replicates per condition. Technical replicates were treated as nested within each independent experiment in the statistical analysis (see Section 2.5).

### 2.5. Statistical Analysis

Data are expressed as scatter plots with bars including mean values with standard deviation (SD). Data were analysed using GraphPad Prism 9 (Statcon, Witzenhausen, Germany). For intergroup comparisons, data were tested for Gaussian distribution and analysed by one-way ANOVA followed by Bonferroni’s multiple comparison test for parametric data or Kruskal-Wallis test with Dunn’s multiple comparison test for non-parametric data. *p*-values < 0.05 were considered as statistically significant. For Seahorse experiments, a nested one-way ANOVA was applied to account for technical replicate wells nested within each independent biological experiment, followed by Bonferroni’s multiple comparisons test. The number of technical and biological replicates and the statistical test applied for each figure are summarised in Supplementary Tables S1 and S2, and exact *p*-values for all comparisons are provided in Supplementary Table S3.

## 3. Results

### 3.1. Hyperoxia-Induced OPC Degeneration and Reduced Proliferation Are Attenuated by hMSC Treatment

**Figure 2:**
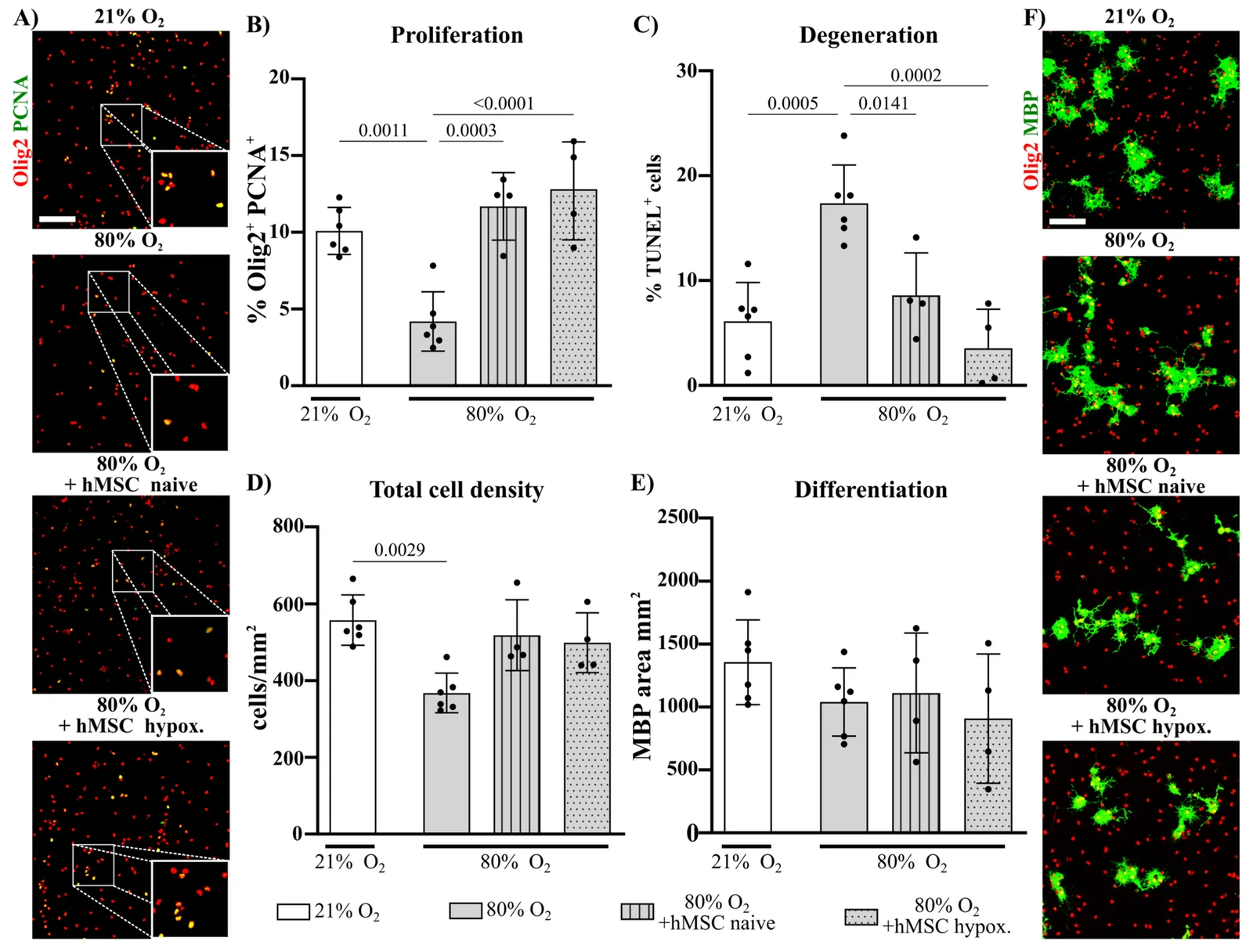
Naive and hypoxic hMSCs protect immature oligodendrocytes against hyperoxia-induced proliferation failure and degeneration. At day 3 in culture, immature oligodendrocytes were exposed to hyperoxia (80% O_2_) for 8 h and co-cultured with naive or hypoxic hMSCs. After a recovery period of 48 h, immature oligodendrocytes were analysed regarding proliferation, degeneration and differentiation. (**A**) Representative images of immunofluorescence staining of immature oligodendrocytes (PCNA green, Olig2 red). (**B**) Quantification of proliferating oligodendrocytes (PCNA^+^/Olig2^+^) in % of all oligodendrocytes. (**C**) Degeneration was assessed by TUNEL staining in combination with Olig2. (**D**) Total cell density of Hoechst^+^ cells was determined. (**E**) The spontaneous differentiation of oligodendrocytes was investigated by MBP staining, measuring the MBP^+^ area. (**F**) Representative images of MBP^+^/Olig2^+^ staining. Scale bar: 100 µm. Data are mean values from 8 images per group per independent experiment (corresponding to an independent cell preparation), and each individual data point presents one independent experiment, n = 4–6 independent experiments per condition. One-way ANOVA followed by a Bonferroni post hoc test (**B**,**C**,**E**) and Kruskal-Wallis test with Dunn’s multiple comparison test (**D**).

Previous studies have shown that immature oligodendrocytes, the predominant population in preterm born infants, are highly sensitive to hyperoxia [4,18,19,25,50,51]. Therefore, we assessed oligodendrocyte viability using the following staining: Olig2/PCNA for proliferation (Figure 2A,B) and Olig2/TUNEL for degeneration (Figure 2C). Exposure to hyperoxia (80% O_2_) for 8 h, followed by a 48 h recovery period (Figure 1A), resulted in a significant reduction of oligodendrocyte proliferation compared to the normoxia control (21% O_2_) (Figure 2A,B). This effect was ameliorated by naive as well as hypoxic hMSCs, while no differences were observed between both hMSC groups (Figure 2A,B). Furthermore, hyperoxia-induced degeneration was reduced by both naive and hypoxic hMSCs (Figure 2C). Reduced OPC proliferation and increased degeneration induced by hyperoxia was also associated with a pronounced reduction of cell density, while cell densities in co-cultures with naive and hypoxic hMSCs were comparable to control OPCs (Figure 2D). Notably, hMSC treatment (naive and hypoxic) under normoxic conditions did not affect proliferation (Supplementary Figure S2A,C), degeneration (Supplementary Figure S2D) and total cell density (Supplementary Figure S2E). Since neonatal hyperoxia has been identified as a risk factor for myelination deficits [28,52], we further assessed myelination capacity of oligodendrocytes in co-staining of Olig2 and MBP (Figure 2E,F). Interestingly, MBP expression was neither modulated by hyperoxia alone nor by either naive or hypoxic hMSC treatment (Figure 2F). Furthermore, we did not observe significant modulations of MBP expression by naive or hypoxic hMSCs under normoxic (21%O_2_) conditions (Supplementary Figure S2B,F).

### 3.2. Hyperoxia-Induced Alterations in Proliferation, Degeneration, and Dendritic Area of Hippocampal Neurons Are Ameliorated by Hypoxic hMSCs

**Figure 3:**
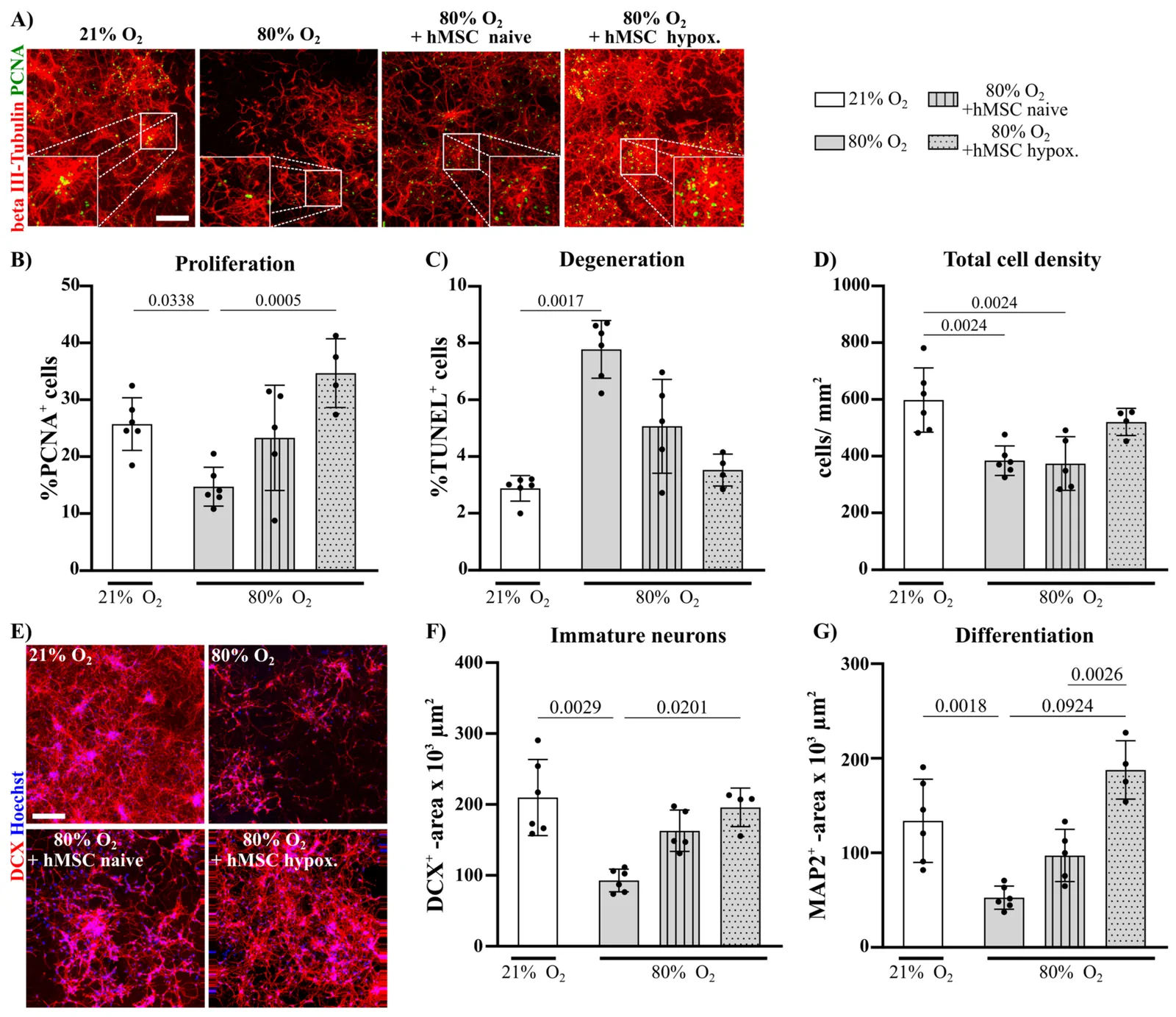
Degeneration, reduced proliferation, and impaired differentiation in hippocampal neurons following hyperoxia are mitigated particularly by hypoxic hMSCs. On day 5 in culture, hippocampal neurons were exposed to hyperoxia (80% O_2_) for 8 h followed by a 48 h recovery phase in non-direct co-culture with naive or hypoxic-preconditioned hMSCs. (**A**) Representative images of betaIII-Tubulin (red)/PCNA (green) staining of proliferating immature hippocampal neurons. (**B**) Quantification of percentages of proliferating hippocampal neurons. (**C**) Quantification of degenerating TUNEL+ cells. (**D**) Total cell density of Hoechst^+^ cells was determined. (**E**) Representative images of immature hippocampal neurons detected by DCX (red). (**F**) Quantification of the DCX^+^ area of immature hippocampal neurons. (**G**) Determination of differentiation of hippocampal neurons analysed by measuring the MAP2^+^ area. Scale bar: 100 µm. Data are mean values from 8 images per group for each independent experiment, and each individual data point presents one independent experiment. n = 4–6 independent experiments. One-way ANOVA followed by a Bonferroni post hoc test (**B**,**D**,**G**) and Kruskal–Wallis test with Dunn’s multiple comparison test (**C**,**F**).

Hyperoxia causes widespread neuronal apoptosis affecting cortical areas, white matter tracts and the hippocampus [15,17,53]. Based on in vivo evidence that neonatal hyperoxia leads to reduced proliferation and neurogenesis, as detected by DCX expression in hippocampal regions, and changes in hippocampal structure [16,17], we investigated the proliferative capacity and degeneration of hippocampal neurons exposed to hyperoxia after a recovery period of 48 h. Proliferation, assessed by PCNA/betaIII-tubulin co-staining (Figure 3A), was significantly reduced by approximately 40% but was improved by hypoxic-preconditioned hMSC treatment (Figure 3B). In addition, hyperoxia-induced degeneration, detected by TUNEL/betaIII-tubulin-positive cells, was slightly improved, particularly by hypoxic-preconditioned hMSCs, though not reaching statistical significance (*p* = 0.0664, Figure 3C). Hyperoxia-induced degeneration and reduced proliferation were associated with a significant reduction in total cell density (Figure 3D). Notably, while naive hMSC treatment did not statistical significantly modulate proliferation, degeneration and total cell density, more pronounced effects were observed with hypoxic-preconditioned hMSCs (Figure 3A–C), revealed by increased total cell numbers, which did not differ from untreated control cells (Figure 3D). Similar to oligodendrocytes, both naive and hypoxic hMSCs had no effect on hippocampal neuron proliferation, degeneration and total cell density under normoxic conditions (Supplementary Figure S3A–D). In vitro modulation of the number of DCX-positive hippocampal neurons revealed that 8 h of hyperoxia followed by 48 h recovery significantly reduced DCX^+^ immature neurons, which was statistically significantly increased by hypoxic-preconditioned hMSCs (Figure 3E,F). The differentiation capacity of hippocampal neurons, assessed by the MAP2-positive area, was also significantly reduced by hyperoxia, while co-culture with hypoxic-preconditioned hMSCs resulted in increased MAP2 expression after hyperoxia exposure (Figure 3G). Notably, neither naive hMSCs nor hypoxic hMSC treatment showed an effect on immature neurons and differentiation under normoxic conditions (Supplementary Figure S3E–G).

### 3.3. Naive and Hypoxic hMSCs Improve Impaired Dendritic Morphology in Hippocampal Neurons

**Figure 4:**
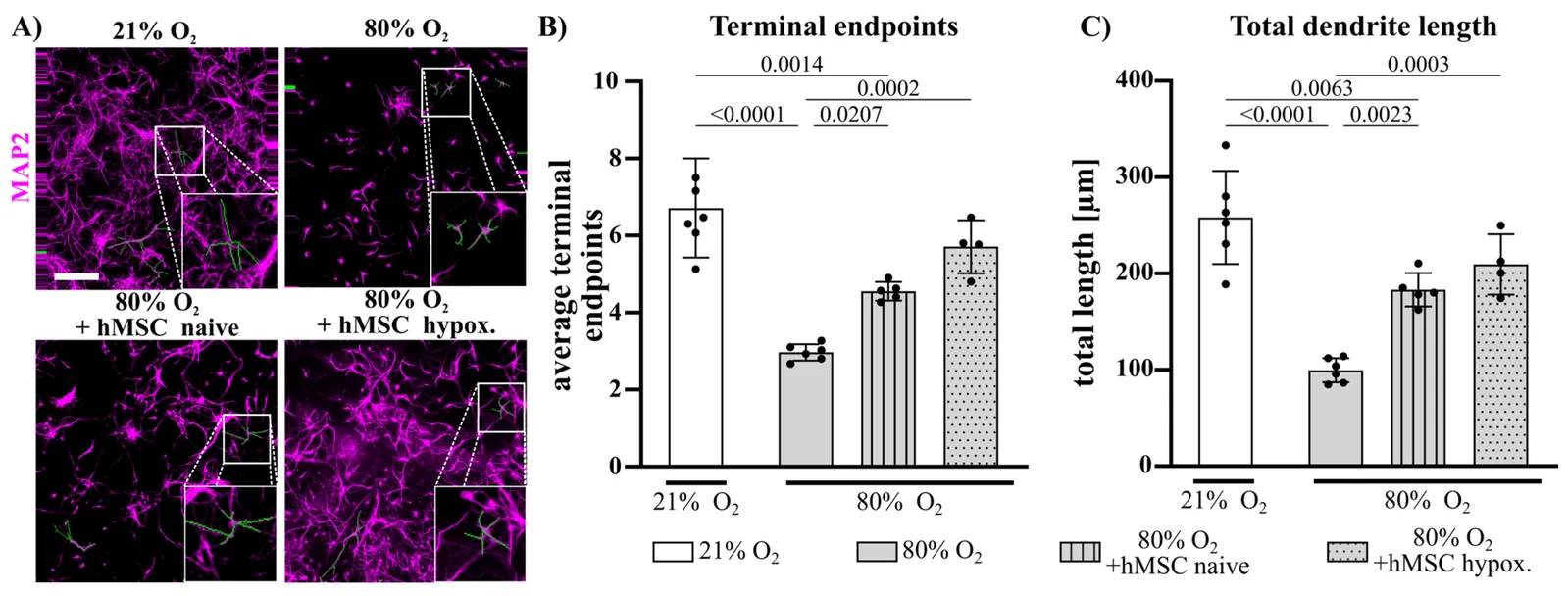
Hyperoxia-induced structural changes of hippocampal neurons are ameliorated by naive and hypoxic-preconditioned hMSCs. On day 5 in culture, hippocampal neurons were exposed to hyperoxia (80% O_2_) for 8 h followed by a 48 h recovery phase in non-contact co-cultures with naive or hypoxic hMSCs. (**A**) Representative images of MAP2^+^ (purple) hippocampal neurons. Green lines in the insets, showing magnified crops of the original images, demonstrate analyses of terminal endpoints and total lengths. (**B**) Quantification of terminal endpoints. (**C**) Total dendrite length of MAP2^+^ hippocampal neurons. Scale bar:100 µm. Individual data points show mean values of 40 cells per group for each independent experiment, n = 4–6 independent experiments. One-way ANOVA followed by a Bonferroni post hoc test.

To determine whether the reduction in the dendrite area was related to changes in dendrite morphology, the number of terminal endpoints and total dendrite length was quantified [54] (Figure 4A). Hyperoxia revealed a reduced number of terminal endpoints and dendrite length, which was statistical significantly ameliorated by both naive and hypoxic-preconditioned hMSCs (Figure 4B,C). No impact of naive and hypoxic-preconditioned hMSCs was observed under normoxic conditions (Supplementary Figure S4A–C).

### 3.4. Naive and Hypoxic hMSCs Restore Mitochondrial Respiration in Oligodendrocytes and Hippocampal Neurons Following Hyperoxic Exposure

**Figure 5:**
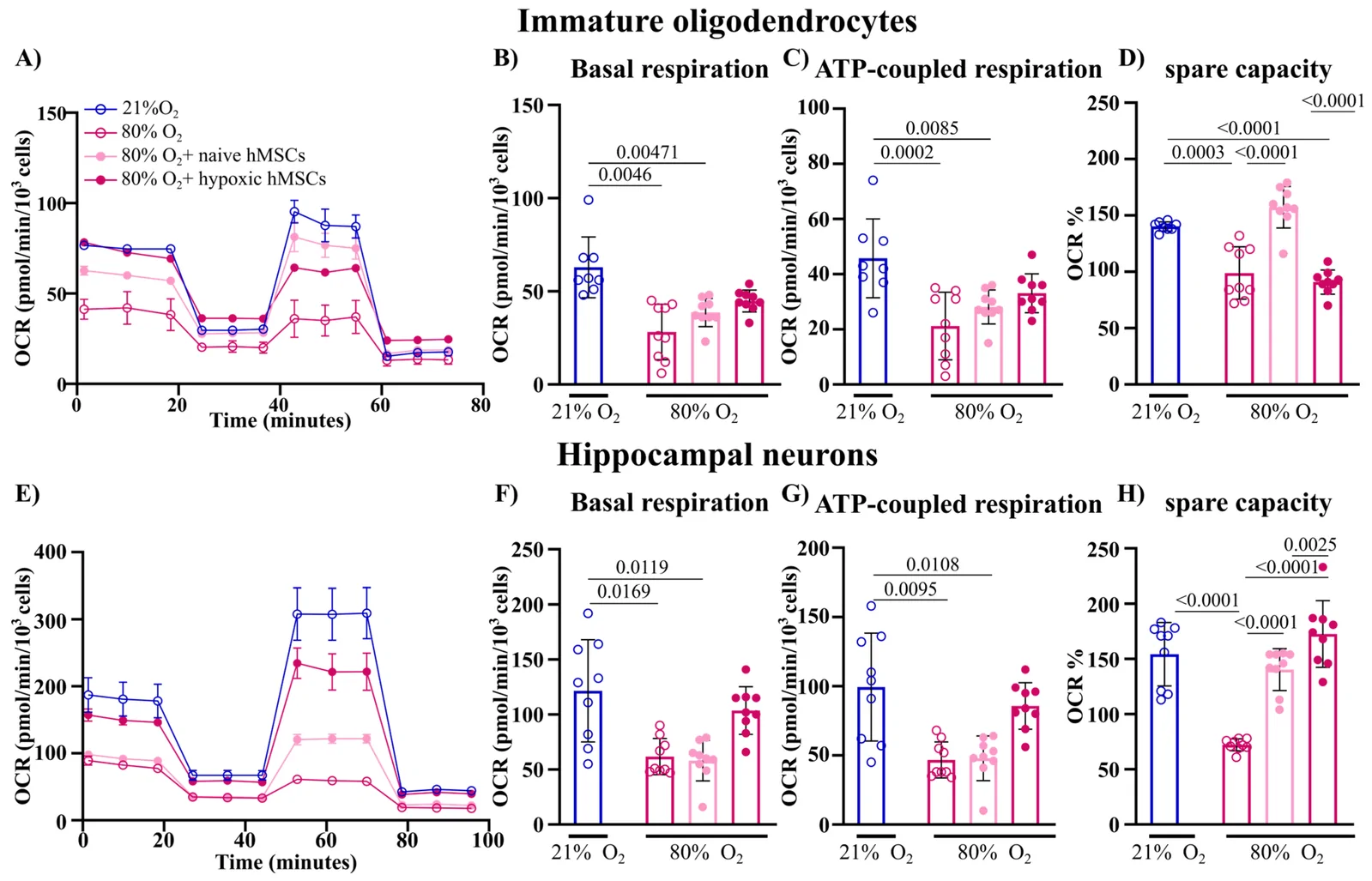
Impaired mitochondrial respiration is improved by naive and hypoxic-preconditioned hMSCs in a target cell type-dependent manner. Oxygen consumption rates of immature oligodendrocytes (**A**–**D**) as well as hippocampal neurons (**E**–**H**) after hyperoxia and 48 h recovery were measured and analysed according to the manufacturer’s guidelines from Agilent for Seahorse XF Cell Mito Stress Kit. **A**) Seahorse oxygen consumption measurement allows differentiation between basal and ATP-linked mitochondrial respiration as well as investigation of the spare capacity (Supplementary Figure S1). Oxygen consumption rate curves (**A**,**E**) show averaged real-time cell responses to oligomycin, FCCP and rotenone-antimycin (Rot/AA). Quantification of basal (**B**,**F**), ATP-linked (**C**,**G**) respiration and spare capacity (**D**,**E**) as a measure of metabolic flexibility. Individual data points present data from 3 independent experiments, each with three technical replicates (9 points total per group). Statistical analysis was performed using a nested one-way ANOVA followed by Bonferroni’s multiple comparisons test, treating technical replicates as nested within each independent biological experiment; n = 3 independent experiments per group was used as the statistical unit.

Previous studies analysing hyperoxia in brain tissue samples and lung epithelial cells have demonstrated that hyperoxia leads to an immediate impairment in mitochondrial respiration [55,56]. However, whether mitochondrial respiration in oligodendrocytes or hippocampal neurons is also affected in the recovery phase after hyperoxia remains unclear. Therefore, we examined oligodendrocyte mitochondrial respiration using Seahorse oxygen consumption rate (OCR) measurements (Figure 5A). We sequentially applied an ATP-synthase blocker (oligomycin), an electron transport chain (ETC) uncoupler (FCCP), and a mitochondrial respiration blocker mixture (rotenone/antimycin A, Rot/AA), allowing the assessment of basal respiration, ATP-linked respiration, and spare capacity (Supplementary Figure S1).

Hyperoxia-induced reduction in the OCR and ATP-linked respiration of oligodendrocytes were not significantly improved by naive and hypoxic-preconditioned hMSCs (Figure 5A–C). In contrast, hyperoxia-induced reduction in spare capacity was significantly restored to control level by naive hMSC treatment (Figure 5D). For hippocampal neurons, we observed a similar reduction in OCR and ATP-linked respiration in hyperoxia-treated cells, which was less pronounced in co-culture with hypoxic-preconditioned hMSCs, while co-culture with naive MSCs had no impact (Figure 5E–G). In contrast, both naive and hypoxic-preconditioned hMSCs significantly improved impaired spare capacity (Figure 5H). For both oligodendrocytes and hippocampal neurons, none of the two hMSC treatments had an impact on mitochondrial respiration (basal, ATP-linked and spare capacity) under normoxic conditions (Supplementary Figure S5A–F).

## 4. Discussion

Neonatal hyperoxia leads to grey and white matter damage in vivo and in vitro [13,15,17,25,57], which is closely associated with the development of EoP. Particularly, immature oligodendrocytes and hippocampal neurons are very susceptible to non-physiological oxygen concentrations, including hyperoxia and hypoxia [38,58,59,60]. So far, no causal therapy exists for EoP [61]. MSC therapy has been identified as a promising candidate due to its previously described neuroprotective effects in both adult and neonatal neurodegenerative diseases [33,34,36,62]. Furthermore, hypoxic preconditioning of hMSCs has been shown to enhance their neuroprotective efficacy [40,41]. In the present study, we analysed the impact of naive versus hypoxic-preconditioned hMSCs on immature oligodendrocytes and hippocampal neurons during the recovery phase after 8 h of hyperoxia. Using a non-contact co-culture system [47], we demonstrated that hyperoxia-induced OPC, hippocampal neuron degeneration and mitochondrial dysfunction can be restored by treatment with naive and hypoxic-preconditioned hMSCs. Notably, while therapeutic effects on oligodendrocytes were comparable between both hMSC cell types, hypoxic preconditioning of MSCs provided a statistically significant advantage compared to naive hMSCs in co-cultures with hippocampal neurons.

While the duration and strength of hyperoxia vary widely in in vivo models [13,38,50,63,64,65], conditions used in cell culture studies, mainly involving primary immature oligodendrocytes, are more standardised [38]. Most studies use 80% hyperoxia for up to 24 h, resulting in acute degeneration, reduced proliferation, and impaired differentiation of oligodendrocytes [13,14,25]. The concentration of 80% O_2_ was chosen to model the marked increase in oxygen exposure after preterm birth, when oxygen tension rises around fourfold compared to the intrauterine environment [4,66]. Nevertheless, this gas-phase concentration should not be interpreted as a direct surrogate for a corresponding rise in arterial or tissue oxygen tension in vivo. Buffering mechanisms of haemoglobin are absent in cell culture, where oxygen in the medium is governed by diffusion through the medium boundary layer and by cell density and consumption, without oxygen-carrying proteins to dampen the rise. Therefore, cells in vitro are exposed to a supraphysiological, unbuffered, and temporally distinct oxygen challenge relative to the in vivo situation [67]. The 80% concentration is therefore best understood as an empirically validated exposure threshold capable of reproducing the relevant injury phenotype, rather than a literal quantitative model of arterial hyperoxia. We reduced the duration of hyperoxia to 8 h to induce a mild but persistent effect that enables assessment over a 48 h recovery phase to dissect the impact of naive and hypoxic-preconditioned hMSCs. Despite shorter hyperoxia duration, our results are consistent with previous studies using 80% hyperoxia for 24 h [14,25,68], revealing a significant increase in degeneration and reduced proliferation of OPCs following 48 h recovery phase after 8 h of hyperoxia. This may be partly related to the absence of a medium change throughout the experiment. Interestingly, similar findings were observed in a recent in vitro study with human umbilical vein endothelial cells (HUVECs), which were exposed to oxygen-glucose deprivation (OGD) followed by reoxygenation [69]. In this study, HUVECs demonstrated reduced cell viability and increased degeneration after 48 h of reoxygenation, which was significantly attenuated with hMSC treatment [69].

EoP is characterised by hypomyelination, and prior in vitro studies have demonstrated that 24 h hyperoxia reduces expression of myelin basic protein (MBP) [13,38]. Interestingly, 8 h of hyperoxia in our study did not result in significant changes in MBP expression. In addition to shorter hyperoxia durations, differences might be explained by different culture conditions. While in the previous study, differentiation was actively induced by the addition of T3 (triiodthyronine) and CNTF (ciliary neurotrophic factor) after 24 h of hyperoxia [13], in the present work, we were interested in the onset of spontaneous myelination, which may explain the lack of modulation in MBP levels. Moreover, it has been reported that the proliferation and differentiation of rat oligodendrocytes are influenced by cell density [70]. Given that lower cell density results in reduced proliferation and increased differentiation [70], the significant reduction in cell density due to hyperoxia observed in the present work might explain the reduced proliferation and no modulation of MBP expression. Future studies should assess additional oligodendrocyte maturation markers such as O4, A2B5 or APC/CC1 to improve the understanding of oligodendrocyte differentiation in this context. Taken together, these findings indicate that even 8 h of hyperoxia followed by a 48 h recovery period is sufficient to impair OPC viability. Treatment with both naive and hypoxic-preconditioned hMSCs significantly attenuated hyperoxia-induced degeneration and restored OPC proliferation.

In addition to oligodendrocytes, neurons exhibit high susceptibility to hyperoxia, as demonstrated in various in vivo models of hyperoxia to induce preterm brain injury or bronchopulmonary dysplasia (BPD) [16,17,31,71]. For example, exposure to 80% oxygen for either 24 or 48 h leads to increased apoptosis and a reduced proliferation of immature neurons [17]. Moreover, chronic exposure to hyperoxia (85% O_2_ from P0 to P14), as used in experimental BPD models, has been shown to decrease hippocampal neurogenesis, demonstrated by reduced numbers of DCX-positive neurons and an overall reduction in hippocampal structure [16]. These results are consistent with our in vitro findings, showing that 8 h of hyperoxia followed by 48 h recovery leads to increased neuronal apoptosis, reduced proliferation, decreased cell density and reduced DCX^+^-neurons. The decrease in immature neurons may be a consequence of both degeneration and impaired proliferation. Similarly, reductions in the MAP2 area may be a direct consequence due to cell degeneration caused by hyperoxia. Nevertheless, it cannot be excluded that, similarly, as described for oligodendrocytes, reduced cell densities caused by hyperoxia perse impair differentiation independent of hyperoxia, which cannot be disentangled in the present experimental setting.

A previous in vivo study suggests that even short-term neonatal hyperoxia may induce long-lasting dendritic alterations that remain detectable until early adulthood [71]. Dendrite length and branching are well established and commonly used parameters to assess structural integrity and development of neurons independent of myelination in vivo and in vitro [72,73]. Beside neuronal viability, morphological development, including dendrite complexity, is essential for hippocampal function [74,75]. While the majority of studies applied prolonged hyperoxia exposures [16,28,50], in the present work, we demonstrate that even short hyperoxia can lead to significant changes in dendrite morphology, including reduced dendrite area, length, and terminal endpoints, which could be restored by hMSCs.

The protective effect of hMSC therapy in our study did not require direct cell-to-cell contact with their target cells, suggesting a paracrine mode of action of hMSCs. Notably, as shown for the vascular endothelium in stroke models, MSCs can also protect stressed cells through direct contact via gap junctions [76], suggesting MSCs use both direct contact and paracrine signals. In support of this, another non-direct in vitro study revealed that non-contact co-culture of hMSCs with oligodendrocytes for 72 h ameliorated inflammation-induced impairment of oligodendrocyte differentiation and myelination [77]. In the latter study, oligodendrocytes were initially exposed to conditioned medium derived from lipopolysaccharide-stimulated microglia instead of hyperoxia prior to non-contact culture with hMSCs, and differentiation was actively induced by addition of CNTF and T3 prior to co-culture [77]. The therapeutic and paracrine mode of MSC-derived extracellular vesicles (EV) is well described in different in vivo models of perinatal brain injury induced by systemic inflammation and/or hypoxia–ischemia [78,79,80]. Across different injury models, MSC-EVs restored impaired oligodendrocyte differentiation and myelination [78,79,81]. With regard to hyperoxia-induced perinatal brain injury, far less is known. In a BPD model with chronic hyperoxia exposure, intratracheal MSC-EV treatment did not only ameliorate lung injury but also improved brain neurogenesis, reflected by enhanced neurosphere formation [16]. Though it is well accepted that the therapeutic effects of MSC treatment are mediated through paracrine mechanisms [32,82,83,84], molecular details are still unclear. With regard to immature oligodendrocytes and neurons, brain-derived neurotrophic factor (BDNF) seems a relevant candidate, as a key player in neuronal and oligodendrocyte survival and growth, but also important for dendrite development [84,85,86]. Therefore, future research should focus on investigating the molecular landscape of MSC-mediated therapeutic signalling pathways.

Even though the pathophysiology of EoP is quite well understood at the cellular level, metabolic cell responses are less explored. Mitochondrial damage, inflammation and reactive oxygen species induced by hyperoxia or hypoxia are thought to be critically involved in the development of EoP [4,38,87]. Most probably due to the insufficient antioxidant defence system of the preterm brain, immature oligodendrocytes and neurons with immature mitochondria are very sensitive to hyperoxia-induced oxidative stress [4,16,27,53,88]. Mitochondrial immaturity may result in impaired ROS neutralisation [55,89], leading to oxidation of proteins, lipids and DNA and finally inducing cell death [25,90]. Chronic hyperoxia exposure in mice from P2 to P14 led to altered hippocampal mitochondrial respiration, particularly basal and ATP-linked respiration [55], although the exact cell source remains unclear. In the present work, we demonstrate that 8 h of hyperoxia followed by 48 h of recovery results in significantly reduced basal and ATP-linked respiration, associated with decreased spare capacity in immature oligodendrocytes and neurons. These results suggest reduced metabolic cell fitness and flexibility to respond to an energetic demand and maximal respiration. Importantly, non-contact co-culture with hMSCs reversed these metabolic disturbances in OPCs and immature neurons, a so-far unknown mechanism of action of MSCs in the setting of immature neural cell degeneration. Even though speculative, therapeutic effects of hMSCs in the present work might be related to mitochondrial transfer from stem cells [43,91,92]. While the precise signals remain unclear, various studies have shown that mitochondrial transfer by MSCs can influence processes, such as proliferation, apoptosis, differentiation, migration, and responses to stress, inflammation, and metabolism [93,94,95]. Tseng et al. showed in vitro that stem cells can transfer healthy mitochondria to oxygen-damaged neurons, improving neuronal metabolism [91]. Hyperoxia induces oxidative stress, damaging both immature oligodendrocytes and neurons, and mitochondria, which in turn release signals that may trigger mitochondrial transfer from stem cells [91,96,97]. Interestingly, even though mitochondrial transfer is believed to depend on cell–cell contact, transfer via extracellular vesicles appears a possible route, which would need further investigation in future studies [43].

We found that hypoxic preconditioning of hMSCs had a significant advantage over naive hMSCs in co-cultures with immature hippocampal neurons. This was reflected by increased cell viability but also improved mitochondrial respiration. Previous studies have already shown that hypoxic preconditioning (2–5% O_2_) can maintain the homogeneity and differentiation potential of MSCs and delay cell senescence while increasing the expression of MSC chemokine receptors [39,40,41]. Additionally, hypoxic preconditioning enhances MSC migration, proliferation and survival, effects that were linked to the glucose-regulated protein 78 (GRP78)/Akt signalling pathway [39,40,98]. Our findings are supported by a study using hypoxic-preconditioned olfactory mucosa stem cells, showing improvements in mitochondrial function and reduced ROS levels in SH-SY5Y neurons after OGD and 24 h reperfusion, compared to naive stem cells [39]. In a model of neonatal hypoxic–ischemic brain injury, hypoxic MSCs demonstrated greater neuroprotective effects associated with increased migratory capacity [47]. Furthermore, in a hindlimb ischemia model, hypoxic-preconditioned MSCs modulated stress and apoptosis-related signals more effectively than naive cells [98], and transplantation of hypoxic-preconditioned MSCs led to increased angiogenic cytokine expression in damaged tissue [98,99]. These cellular responses following hypoxic preconditioning were associated with higher secretion levels of growth factors, including, for example, vascular endothelial growth factor (VEGF), BDNF and insulin-like growth factor (IGF), all crucial for neuroprotection [99]. In a traumatic brain injury model, the secretome from hypoxic-preconditioned MSCs significantly improved brain recovery compared to naive stem cells [100]. Together, these findings could explain why hypoxic-preconditioned stem cells provide a stronger therapeutic effect on hyperoxia-damaged neurons.

In the present study, we investigated a single, standardised hyperoxic insult (80% O_2_ for 8 h), which represents an acute experimental insult and cannot capture the complexity of risk factors associated with the development of EoP, including fluctuating oxygenation, altered perfusion and comorbidities such as infection and bronchopulmonary dysplasia (BPD), typically observed in preterm infants with EoP. Furthermore, interactions with other cell types, e.g., microglia or astrocytes, and the vasculature cannot be adequately assessed. hMSCs used in the present work were derived from a single donor, and donor variability will need to be addressed in future studies. Future work should further validate these therapeutic effects, including analyses of additional cell maturation markers and assessment of the potential of hMSCs-derived extracellular vesicles, both in vitro and in established in vivo models of hyperoxia-induced EoP models and combinations with other noxious factors, e.g., maternal inflammation [28]. Furthermore, our in vitro model does not allow the assessment of neuron–oligodendrocyte interactions or functional myelination, as would be possible in co-culture or organotypic slice culture systems [101,102], which should be investigated in future studies using more complex experimental models. Lastly, the direct co-culture setup in the present work primarily models selected intracerebral applications rather than systemic administration, which is a strength in the present context. In the absence of a blood–brain barrier and of systemic effects, the observed responses can be attributed more directly to the cell types investigated, and effects are most likely mediated by secreted soluble factors and/or extracellular vesicles. Nevertheless, where these factors ultimately act in vivo, and via which routes, is an important question that future work will need to elaborate.

## 5. Conclusions

This study investigated the therapeutic effects of naive and hypoxic-preconditioned human Wharton’s jelly-derived umbilical cord MSCs on immature oligodendrocytes and hippocampal neurons exposed to hyperoxia, a key risk factor for the development of EoP. Hyperoxia impaired dendrite differentiation in neurons and reduced cell viability, proliferation and mitochondrial function in both cell types. While treatment with naive and hypoxic-preconditioned hMSCs restored these disturbances, hypoxic preconditioning of hMSCs revealed stronger therapeutic effects, particularly on hippocampal neurons. MBP expression was not significantly affected by hyperoxia and was, therefore, not modulated by hMSC treatment. Our findings suggest that hypoxic preconditioning enhances the therapeutic potential of hMSCs, possibly through the secretion of beneficial factors and stabilisation of mitochondrial function. These results provide in vitro proof-of-concept evidence supporting the therapeutic potential of hMSCs on two major neural cell types. Further in vivo studies are needed to define optimal dosing, administration routes and therapeutic windows and to verify safety, persistence effects due to myelination, and long-term outcomes before clinical translation can be considered. In addition, molecular mechanisms need to be investigated, including the role of mitochondrial transfer, to further explore the therapeutic potential of MSC therapy.

## Data Availability

The original data shown in this manuscript are available from the corresponding author upon reasonable request

## Supplementary Materials

The following supporting information can be downloaded at: https://www.mdpi.com/article/10.3390/cells15161493/s1. Supplementary Figure S1: Adapted Agilent Seahorse XF Cell Mito Stress Test profile. Supplementary Figure S2: Naive and hypoxic hMSCs have no effect on oligodendrocyte proliferation, degeneration and differentiation under standard culture conditions. Supplementary Figure S3: Naive and hypoxic-preconditioned hMSCs do not modulate hippocampal neuron vitality and differentiation under normoxic conditions (21% O_2_). Supplementary Figure S4: Naive and hypoxic-preconditioned hMSCs do not lead to any structural changes under normoxic conditions (21% O_2_). Supplementary Figure S5: Naive and hypoxic-preconditioned hMSCs do not alter mitochondrial respiration under normoxic conditions (21% O_2_). Supplementary Figure S6: Single-channel images of main and supplementary figures. Supplementary Table S1: Summary of technical and biological replicates and statistical tests applied for each figure in the main manuscript. Supplementary Table S2: Summary of technical and biological replicates and statistical tests applied for each Supplement figure. Supplementary Table S3: Exact *p*-values of main figures. Significant values were highlighted in grey.
