# Supplementary material for: Human Mesenchymal Stromal Cells Attenuate Hyperoxia-Induced Cellular Impairment of Immature Oligodendrocyte and Neurons
Source: Cells. 2026 Aug 19;15(16):1493. doi: 10.3390/cells15161493 (PMC13511089; doi:10.3390/cells15161493)
Supplement: Supplementary file 1 [file cells-15-01493-s001.zip › cells-4444686-supplementary.pdf]

Supplementary Material - Human mesenchymal stromal cells attenuate hyperoxia-induced cellular impairment of immature oligodendrocyte and neurons

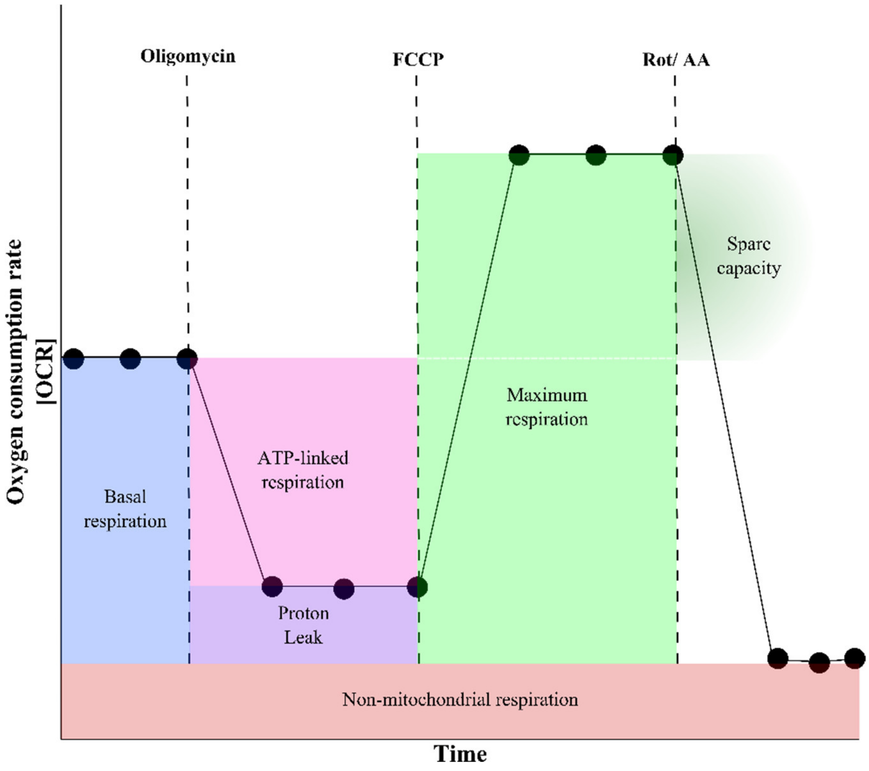

Figure S1. Adapted Agilent Seahorse XF Cell Mito Stress Test profile**.** Oxygen consumption rate [OCR] was measured over a defined time. Investigation of non-mitochondrial respiration, basal respiration, ATP-linked respiration, Proton-Leak, maximal respiration was reached by sequential treating with oligomycin (inhibit ATP-synthase), uncoupler Carbonylcyanide-4 (trifluoromethoxy) phenylhydrazone (FCCP) and rotenone/ antimycin A (Rot/AA, inhibit complex I and III of the mitochondrial electron transfer chain).

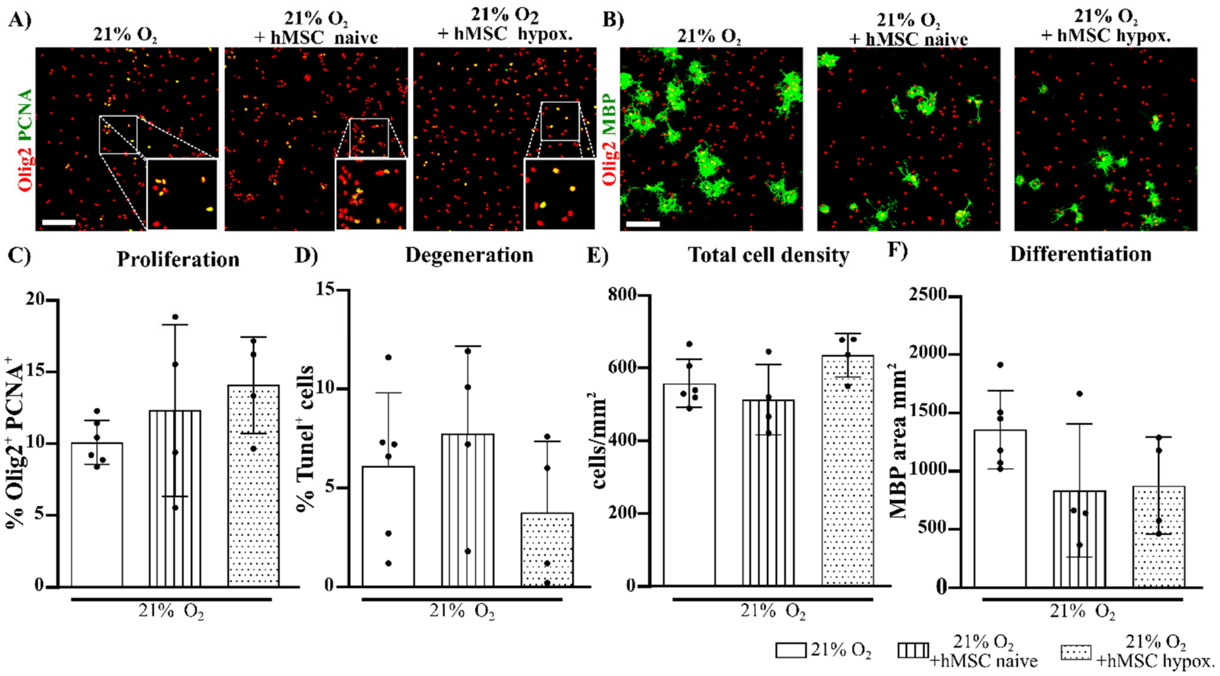

Figure S2. Naive and hypoxic hMSCs have no effect on oligodendrocyte proliferation, degeneration and differentiation under standard culture conditions. At day 3 immature oligodendrocytes were exposed to normoxia (21% O_2) for 8 h and co-cultured with naïve or hypoxic-preconditioned hMSC. After a recovery period of 48 h immature oligodendrocytes were analysed regarding proliferation, degeneration and differentiation. A) Representative images of immunofluorescence staining of proliferating immature oligodendrocytes (PCNA green, Olig2 red). B) Representative images of immunofluorescence staining of differentiated oligodendrocytes (MBP green, Olig2 red). C) Quantification of proliferating oligodendrocytes ($PCNA^+$/ $Olig2^+$) in %. D) Degeneration was assessed by Tunel-staining in combination with Olig2. E) Total cell density of $Hoechst^+$ cells was determined. F) The spontaneous differentiation of oligodendrocytes was investigated by MBP staining, quantifying the MBP^+ area. Scale bar: 100 µm. Data are mean values from 8 images per group for each independent experiment. n= 4-6 independent experiments.

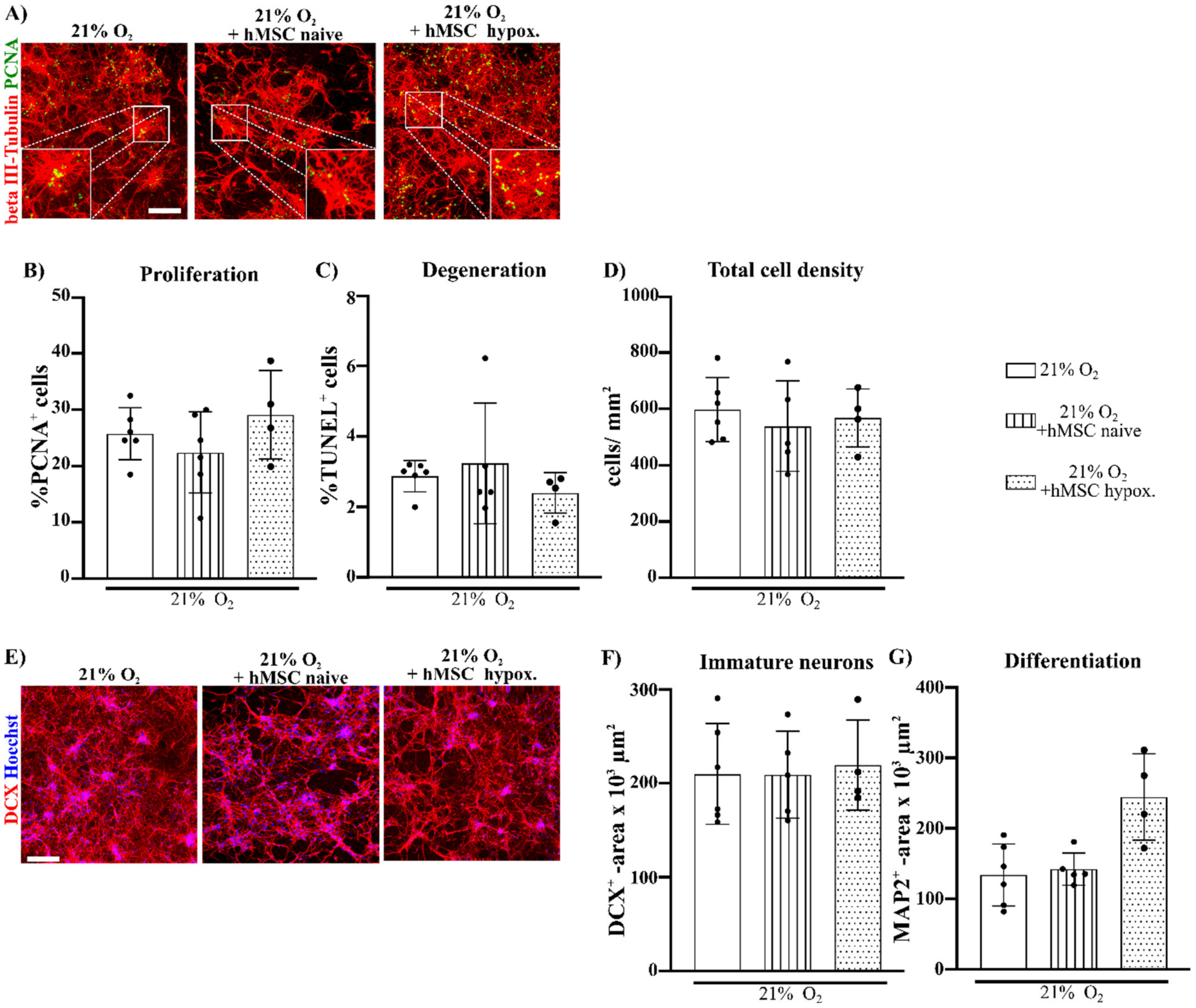

Figure S3. Naive and hypoxic-preconditioned hMSCs do not modulate hippocampal neuron vitality and differentiation under normoxic conditions (21% O_2). At day 5 hippocampal neurons were exposed to normoxia (21% O_2) for 8 h followed by a 48 h recovery phase in non direct co-cultures with naïve or hypoxic-preconditioned hMSCs. A) Representative images of betaIII-Tubulin (red)/ PCNA (green) staining of proliferating immature hippocampal neurons. B) Quantification of percentages of proliferating hippocampal neurons. C) Investigation of degeneration by TUNEL-staining. D) Total cell density of Hoechst$^+$ cells was determined. E) Representative images of immature hippocampal neurons DCX (red). F) Quantification of DCX$^+$ area of immature hippocampal neurons. G) Differentiation of hippocampal neurons analysed by quantification of the MAP2$^+$ area. Scale bar:100 µm. Data are mean values from 8 images per group for each independents experiment. n= 4-6 independent experiments.

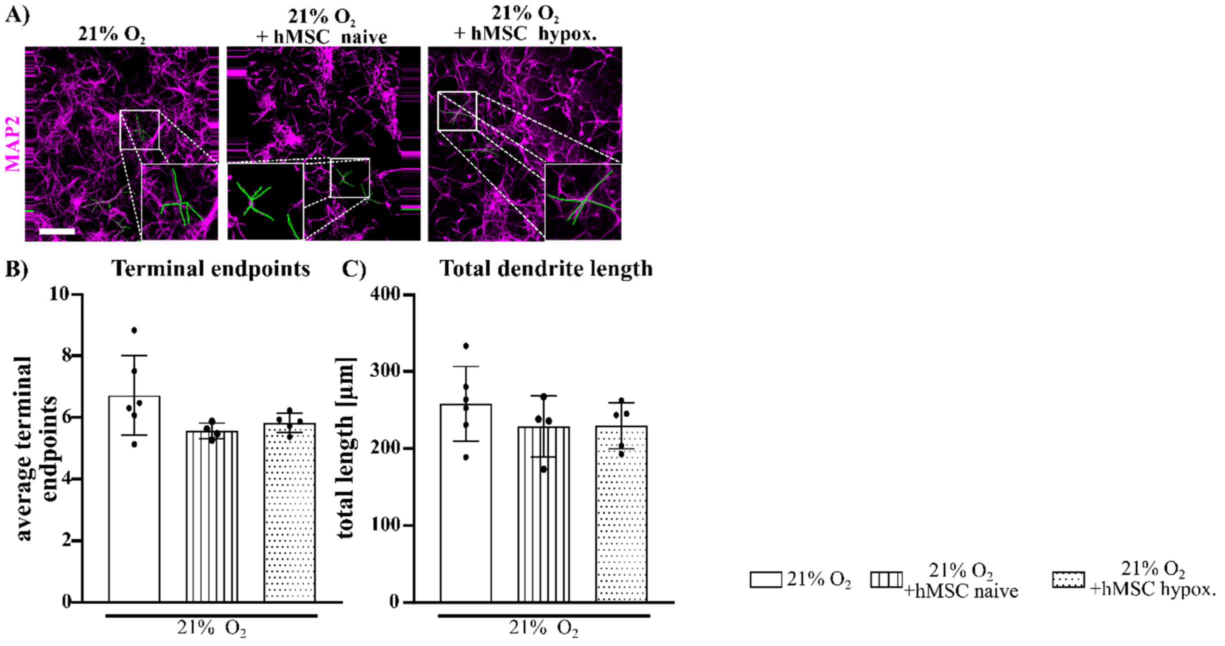

Figure S4. Naive and hypoxic-preconditioned hMSCs do not lead to any structural changes under normoxic conditions (21% O_2). At day 5 hippocampal neurons were exposed to normoxia (21% O_2) for 8 h followed by a 48 h recovery phase in non-direct co-cultures with naive or hypoxic-preconditioned hMSCs. A) Representative images of dendrites, visualized by staining of MAP2 (purple). Green lines in the insets, showing magnified crops of the original images, demonstrate analyses of terminal endpoints and total lengths. C) Determination of total length of MAP2$^+$ dendrites. Scale bar: 100 µm. Data are mean values of 40 cells per group for each independent experiment. n= 4-6 independent experiments.

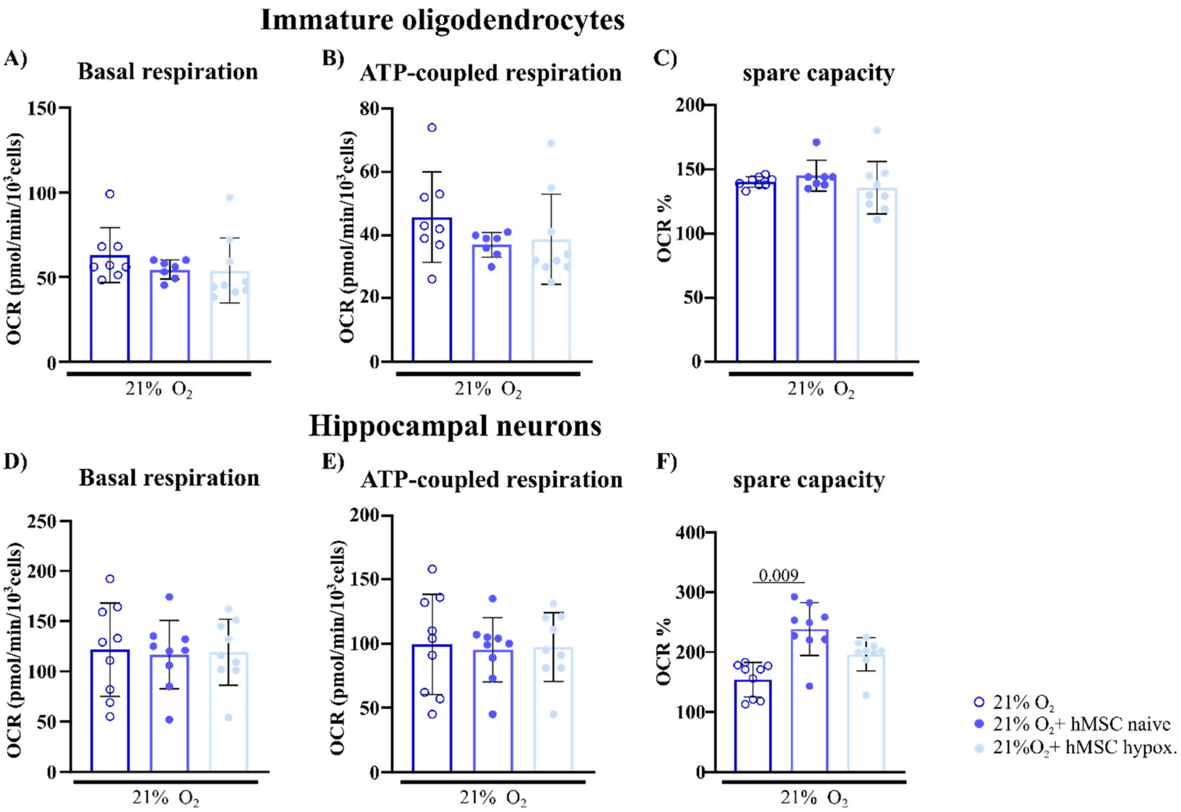

Figure S5. Naive and hypoxic-preconditioned hMSCs do not alter mitochondrial respiration under normoxic conditions (21% O_2). Oxygen consumption rates of immature oligodendrocytes or hippocampal neurons after normoxia and 48 h recovery phase were measured and analysed according manufacturer´s guidelines from Agilent for Seahorse XF Cell Mito Stress Kit. A) Quantification of basal, B) ATP-linked respiration and C) spare capacity as a measure of cell flexibility. D) Quantification of basal, E) ATP-linked respiration and F) spare capacity. Each graph shows all data points from n = 3 independent biological experiments per cell type, each performed with three technical replicates (9 points total per group). Statistical analysis was performed using a nested one-way ANOVA followed by Bonferroni's multiple comparisons test, treating technical replicates as nested within each independent biological experiment; n = 3 independent experiments per group was used as the statistical unit.

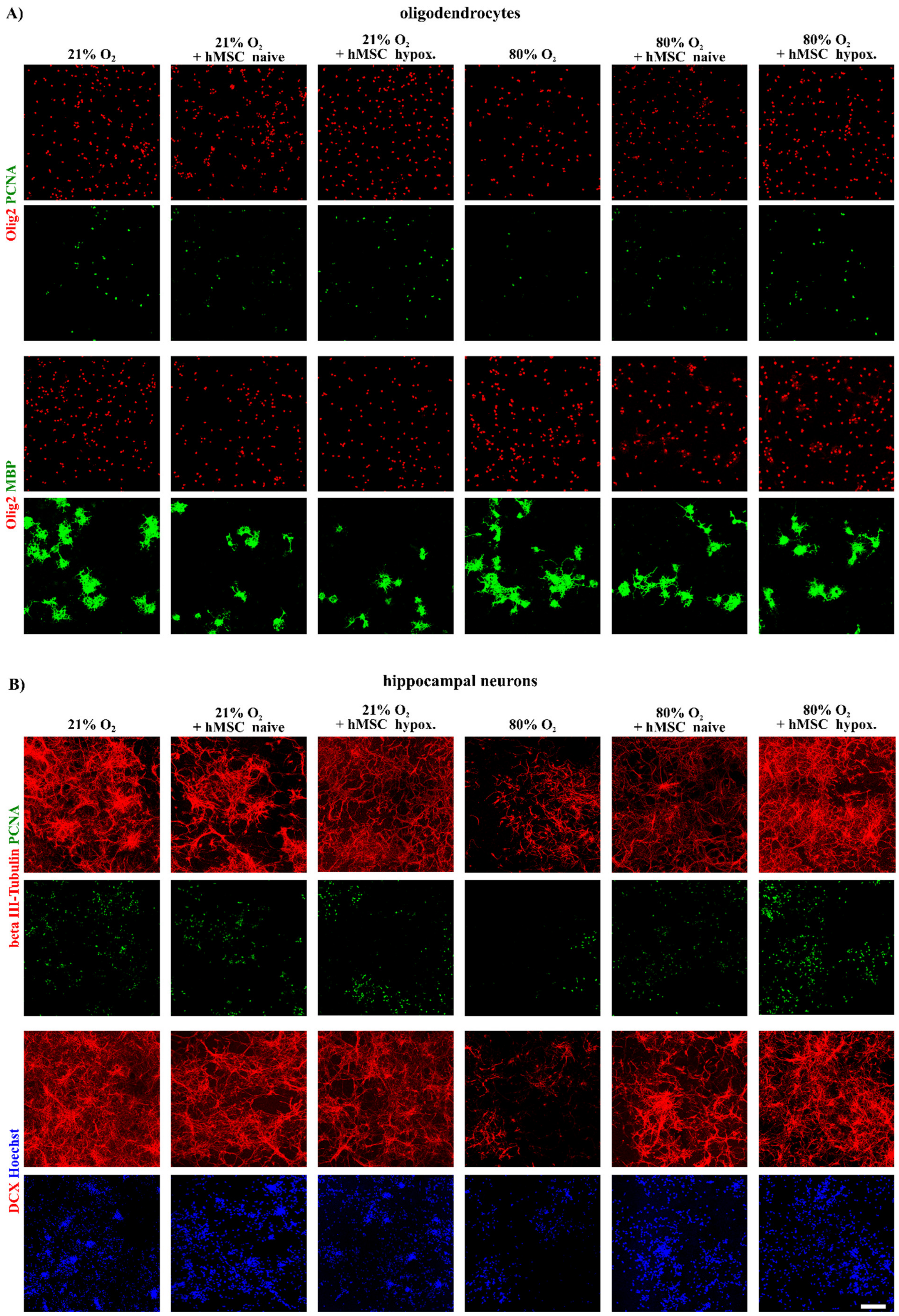
A)
oligodendrocytes
21% O2
21% O2 + hMSC naive
21% O2 + hMSC hypox.
80% O2
80% O2 + hMSC naive
80% O2 + hMSC hypox.
Olig2 PCNA
Olig2 MBP
B)
hippocampal neurons
21% O2
21% O2 + hMSC naive
21% O2 + hMSC hypox.
80% O2
80% O2 + hMSC naive
80% O2 + hMSC hypox.
beta III-Tubulin PCNA
DCX Hoechst

Figure S6. Single-channel images of main and supplementary figures**. A)** Individual fluorescence channels of immature oligodendrocytes across all experimental groups (21% O_2; 21% O_2 + hMSC naive; 21% O_2 + hMSC hypoxic; 80% O_2; 80% O_2 + hMSC naive; 80% O_2 + hMSC hypoxic). Top rows: Olig2 (red) and PCNA (green), used to assess oligodendrocyte proliferation. Bottom rows: Olig2 (red) and MBP (green), used to assess oligodendrocyte differentiation. **B)** Individual fluorescence channels of hippocampal neurons across experimental groups. Top rows: betaIII-tubulin (red) and PCNA (green), used to assess neuronal proliferation. Bottom rows: DCX (red) and Hoechst (blue), used to assess immature neuron density. Scale bar = 100 μm.

Table S1. Summary of technical and biological replicates and statistical tests applied for each figure in the main manuscript.

Figure	Technical replicates	Biological replicates	Statistics	Figure	Technical replicates	Biological replicates	Statistics
Fig. 2B	8 ROIs	4-6	One-Way-ANOVA; post hoc Bonferroni´s multiple comparison test	Fig. 3F	8 ROIs	4-6	Kruskal-Wallis; post hoc Dunns´s multiple comparison test
Fig. 2C	8 ROIs	4-6	One-Way-ANOVA; post hoc Bonferroni´s multiple comparison test	Fig. 3G	8 ROIs	4-6	One-Way-ANOVA; post hoc Bonferroni´s multiple comparison test
Fig. 2D	8 ROIs	4-6	Kruskal-Wallis; post hoc Dunns´s multiple comparison test	Fig. 4B	8 ROIs (5 neurons/ ROI)	4-6	One-Way-ANOVA; post hoc Bonferroni´s multiple comparison test
Fig. 2E	8 ROIs	4-6	One-Way-ANOVA; post hoc Bonferroni´s multiple comparison test	Fig. 4C	8 ROIs (5 neurons/ ROI)	4-6	One-Way-ANOVA; post hoc Bonferroni´s multiple comparison test
Fig. 3B	8 ROIs	4-6	One-Way-ANOVA; post hoc Bonferroni´s multiple comparison test	Fig. 5B	3 Wells	3	Nested one-Way-ANOVA; post hoc Bonferroni´s multiple comparison test
Fig. 3C	8 ROIs	4-6	Kruskal-Wallis; post hoc Dunns´s multiple comparison test	Fig. 5C	3 Wells	3	Nested one-Way-ANOVA; post hoc Bonferroni´s multiple comparison test
Fig. 3D	8 ROIs	4-6	One-Way-ANOVA; post hoc Bonferroni´s multiple comparison test	Fig. 5D	3 Wells	3	Nested one-Way-ANOVA; post hoc Bonferroni´s multiple comparison test

Figure	Technical replicates	Biological replicates	Statistics
Fig. 5F	3 Wells	3	Nested one-Way-ANOVA; post hoc Bonferroni´s multiple comparison test
Fig. 5G	3 Wells	3	Nested one-Way-ANOVA; post hoc Bonferroni´s multiple comparison test
Fig. 5H	3 Wells	3	Nested one-Way-ANOVA; post hoc Bonferroni´s multiple comparison test

Table S2. Summary of technical and biological replicates and statistical tests applied for each Supplement figure.

Figure	Technical replicates	Biological replicates	Statistics	Figure	Technical replicates	Biological replicates	Statistics
Suppl. 2C	8 ROIs	4-6	One-Way-ANOVA; post hoc Bonferroni´s multiple comparison test	Suppl. 2F	8 ROIs	4-6	Kruskal-Wallis; post hoc Dunn´s multiple comparison test
Suppl. 2D	8 ROIs	4-6	One-Way-ANOVA; post hoc Bonferroni´s multiple comparison test	Suppl. 2G	8 ROIs	4-6	One-Way-ANOVA; post hoc Bonferroni´s multiple comparison test
Suppl. 2E	8 ROIs	4-6	One-Way-ANOVA; post hoc Bonferroni´s multiple comparison test	Suppl. 4B	8 ROIs (5 neurons/ ROI)	4-6	One-Way-ANOVA; post hoc Bonferroni´s multiple comparison test
Suppl. 2F	8 ROIs	4-6	One-Way-ANOVA; post hoc Bonferroni´s multiple comparison test	Suppl. 4C	8 ROIs (5 neurons/ ROI)	4-6	One-Way-ANOVA; post hoc Bonferroni´s multiple comparison test
Suppl. 3C	8 ROIs	4-6	One-Way-ANOVA; post hoc Bonferroni´s multiple comparison test	Suppl. 5A	3 Wells	3	Nested one-Way-ANOVA; post hoc Bonferroni´s multiple comparison test
Suppl. 3D	8 ROIs	4-6	Kruskal-Wallis; post hoc Dunns´s multiple comparison test	Suppl. 5B	3 Wells	3	Nested one-Way-ANOVA; post hoc Bonferroni´s multiple comparison test
Suppl. 3E	8 ROIs	4-6	One-Way-ANOVA; post hoc Bonferroni´s multiple comparison test	Suppl. 5C	3 Wells	3	Nested one-Way-ANOVA; post hoc Bonferroni´s multiple comparison test

Figure	Technical replicates	Biological replicates	Statistics
Suppl. 5D	3 Wells	3	Nested one-Way-ANOVA; post hoc Bonferroni´s multiple comparison test
Suppl. 5E	3 Wells	3	Nested one-Way-ANOVA; post hoc Bonferroni´s multiple comparison test
Suppl. 5F	3 Wells	3	Nested one-Way-ANOVA; post hoc Bonferroni´s multiple comparison test

Information Fig. 2, Fig. 3, Figure S2 and Figure S3: For all immunocytochemical analyses, eight randomly selected ROIs were analysed per independent cell preparation. Quantifications from the ROIs were averaged to obtain one value per independent experiment. Each data point represents one independent biological unit.

Information Fig. 4 and Figure S4: For dendritic analyses, eight randomly selected ROIs were analysed per independent cell preparation. Five neurons per ROI were randomly selected and analysed, i.e. 40 neurons per independent experiment; obtained values were averaged to one value per independent experiment. Each data point represents one independent biological replicate.

Information Fig. 5 and Figure S5: For Seahorse analyses, measurements were performed in triplicates per independent experiment. Each data point represents one technical replicate. Statistics were performed using a nested one-way ANOVA, treating wells as nested within each independent experiment.

Table S3. Exact p-values of main figures. Significant values were highlighted in grey.

Figures	Comparison	p value	Figures	Comparison	p value
Fig. 2B	21% O_2 vs 80% O_2	0.0011	Fig. 3B	21% O_2 vs 80% O_2	0.0338
	21% O_2 vs 80% O_2+hMSC naive	>0.9999		21% O_2 vs 80% O_2+hMSC naive	>0.9999
	21% O2 vs 80% O2+hMSC hypox.	>0.9999		21% O2 vs 80% O2+hMSC hypox.	0.2037
	80% O_2 vs 80% O_2+hMSC naive	0.0003		80% O_2 vs 80% O_2+hMSC naive	0.1832
	80% O2 vs 80% O2+hMSC hypox.	>0.9999		80% O2 vs 80% O2+hMSC hypox.	0.0005
	80% O2+hMSC naive vs 80% O2+hMSC hypox.	<0.0001		80% O2+hMSC naive vs 80% O2+hMSC hypox.	0.0714
Fig. 2C	21% O_2 vs 80% O_2	0.0005	Fig. 3C	21% O_2 vs 80% O_2	0.0017
	21% O_2 vs 80% O_2+hMSC naive	>0.9999		21% O_2 vs 80% O_2+hMSC naive	0.5211
	21% O2 vs 80% O2+hMSC hypox.	>0.9999		21% O2 vs 80% O2+hMSC hypox.	>0.9999
	80% O_2 vs 80% O_2+hMSC naive	0.0141		80% O_2 vs 80% O_2+hMSC naive	0.4830
	80% O2 vs 80% O2+hMSC hypox.	0.0002		80% O2 vs 80% O2+hMSC hypox.	0.0797
	80% O2+hMSC naive vs 80% O2+hMSC hypox.	0.4526		80% O2+hMSC naive vs 80% O2+hMSC hypox.	>0.9999
Fig. 2D	21% O_2 vs 80% O_2	0.0029	Fig. 3D	21% O_2 vs 80% O_2	0.0024
	21% O_2 vs 80% O_2+hMSC naive	>0.9999		21% O_2 vs 80% O_2+hMSC naive	0.0024
	21% O2 vs 80% O2+hMSC hypox.	>0.9999		21% O2 vs 80% O2+hMSC hypox.	>0.9999
	80% O_2 vs 80% O_2+hMSC naive	0.1648		80% O_2 vs 80% O_2+hMSC naive	>0.9999
	80% O2 vs 80% O2+hMSC hypox.	0.3905		80% O2 vs 80% O2+hMSC hypox.	0.1344
	80% O2+hMSC naive vs 80% O2+hMSC hypox.	>0.9999		80% O2+hMSC naive vs 80% O2+hMSC hypox.	0.1116
Fig. 2E	21% O_2 vs 80% O_2	>0.9999	Fig. 3F	21% O_2 vs 80% O_2	0.0029
	21% O_2 vs 80% O_2+hMSC naive	0.5535		21% O_2 vs 80% O_2+hMSC naive	0.9039
	21% O2 vs 80% O2+hMSC hypox.	>0.9999		21% O2 vs 80% O2+hMSC hypox.	>0.9999
	80% O_2 vs 80% O_2+hMSC naive	>0.9999		80% O_2 vs 80% O_2+hMSC naive	0.3528
	80% O2 vs 80% O2+hMSC hypox.	>0.9999		80% O2 vs 80% O2+hMSC hypox.	0.0201
	80% O2+hMSC naive vs 80% O2+hMSC hypox.	>0.9999		80% O2+hMSC naive vs 80% O2+hMSC hypox.	>0.9999

Figures	Comparison	p value	Figures	Comparison	p value
Fig. 3G	21% O_2 vs 80% O_2	0.0018	Fig. 5B	21% O_2 vs 80% O_2	0.0046
	21% O_2 vs 80% O_2+hMSC naive	0.4050		21% O_2 vs 80% O_2+hMSC naive	0.0471
	21% O2 vs 80% O2+hMSC hypox.	0.0924		21% O2 vs 80% O2+hMSC hypox.	0.2197
	80% O_2 vs 80% O_2+hMSC naive	0.1786		80% O_2 vs 80% O_2+hMSC naive	0.7292
	80% O2 vs 80% O2+hMSC hypox.	<0.0001		80% O2 vs 80% O2+hMSC hypox.	0.1515
	80% O2+hMSC naive vs 80% O2+hMSC hypox.	0.0026		80% O2+hMSC naive vs 80% O2+hMSC hypox.	>0.9999
Fig. 4B	21% O_2 vs 80% O_2	<0.0001	Fig. 5C	21% O_2 vs 80% O_2	0.0002
	21% O_2 vs 80% O_2+hMSC naive	0.0014		21% O_2 vs 80% O_2+hMSC naive	0.0085
	21% O2 vs 80% O2+hMSC hypox.	0.3545		21% O2 vs 80% O2+hMSC hypox.	0.1048
	80% O_2 vs 80% O_2+hMSC naive	0.0207		80% O_2 vs 80% O_2+hMSC naive	>0.9999
	80% O2 vs 80% O2+hMSC hypox.	0.0002		80% O2 vs 80% O2+hMSC hypox.	0.1552
	80% O2+hMSC naive vs 80% O2+hMSC hypox.	0.02371		80% O2+hMSC naive vs 80% O2+hMSC hypox.	>0.9999
Fig.4C	21% O_2 vs 80% O_2	<0.0001	Fig.5D	21% O_2 vs 80% O_2	0.0003
	21% O_2 vs 80% O_2+hMSC naive	0.0063		21% O_2 vs 80% O_2+hMSC naive	0.0789
	21% O2 vs 80% O2+hMSC hypox.	0.1654		21% O2 vs 80% O2+hMSC hypox.	<0.0001
	80% O_2 vs 80% O_2+hMSC naive	0.0023		80% O_2 vs 80% O_2+hMSC naive	<0.0001
	80% O2 vs 80% O2+hMSC hypox.	0.0003		80% O2 vs 80% O2+hMSC hypox.	>0.9999
	80% O2+hMSC naive vs 80% O2+hMSC hypox.	>0.9999		80% O2+hMSC naive vs 80% O2+hMSC hypox.	<0.0001

Figures	Comparison	p value
Fig. 5F	21% O_2 vs 80% O_2	0.0169
	21% O_2 vs 80% O_2+hMSC naive	0.0119
	21% O2 vs 80% O2+hMSC hypox.	>0.9999
	80% O_2 vs 80% O_2+hMSC naive	>0.9999
	80% O2 vs 80% O2+hMSC hypox.	0.1075
	80% O2+hMSC naive vs 80% O2+hMSC hypox.	0.0724
Fig. 5G	21% O_2 vs 80% O_2	0.0095
	21% O_2 vs 80% O_2+hMSC naive	0.0108
	21% O2 vs 80% O2+hMSC hypox.	>0.9999
	80% O_2 vs 80% O_2+hMSC naive	>0.9999
	80% O2 vs 80% O2+hMSC hypox.	0.0509
	80% O2+hMSC naive vs 80% O2+hMSC hypox.	0.0589
Fig.5H	21% O_2 vs 80% O_2	<0.0001
	21% O_2 vs 80% O_2+hMSC naive	>0.9999
	21% O2 vs 80% O2+hMSC hypox.	0.0683
	80% O_2 vs 80% O_2+hMSC naive	<0.0001
	80% O2 vs 80% O2+hMSC hypox.	<0.0001
	80% O2+hMSC naive vs 80% O2+hMSC hypox.	0.0025
